# Amniotic fluid stem cells and the cell source repertoire for non-invasive prenatal testing

**DOI:** 10.1007/s12015-021-10228-5

**Published:** 2021-08-12

**Authors:** Margit Rosner, Thomas Kolbe, Viktor Voronin, Markus Hengstschläger

**Affiliations:** 1 Institute of Medical Genetics, Center for Pathobiochemistry and Genetics, Medical University of Vienna, Währinger Strasse 10, 1090 Vienna, Austria; 2 Biomodels Austria, University of Veterinary Medicine Vienna, Vienna, Austria; 3 Department IFA Tulln, University of Natural Resources and Life Sciences, Tulln, Austria

**Keywords:** Amniotic fluid stem cells, Cell-free fetal DNA, Non-invasive prenatal testing, Pregnancy-associated progenitor cells

## Abstract

Cell-free fetal DNA (cffDNA)-based non-invasive prenatal testing (NIPT) is considered to be a very promising screening tool for pregnant women with an increased risk of fetal aneuploidy. Already millions of women worldwide underwent NIPT. However, due to the observed false-positive and false-negative results, this screening approach does not fulfil the criteria of a diagnostic test. Accordingly, positive results still require risk-carrying invasive prenatal testing, such as amniocentesis or chorionic villus sampling (CVS), for confirmation. Such hurdles need to be overcome before NIPT could become a diagnostic approach widely used in the general population. Here we discuss new evidence that besides the placenta amniotic fluid stem cells (AFSCs) could also represent an origin of cffDNA in the mother’s blood. A comprehensive picture of the involved cell source repertoire could pave the way to more reliable interpretations of NIPT results and ameliorate counselling of advice-seeking patients.

**Graphical abstract:**

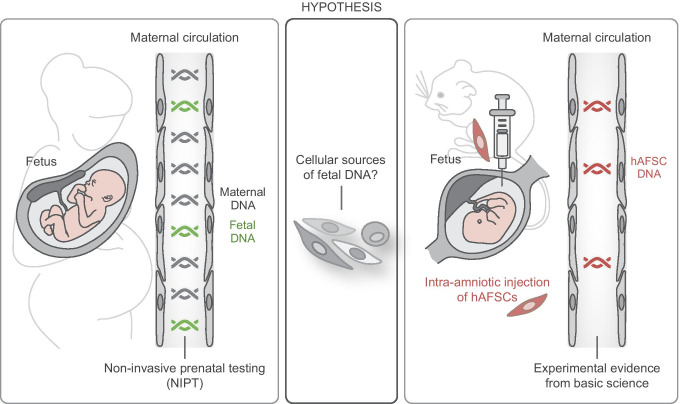

Over two decades ago, the identification of fetal DNA in the maternal circulation sparked a revolution in prenatal diagnosis [1]. Prenatal genetic testing has witnessed a progressive evolution from the invasive amniocentesis and CVS, which are still the diagnostic gold standard but carry a risk of miscarriage, to cffDNA-based NIPT. Since 2011, in many regions of the world NIPT is routinely offered to pregnant women with increased risk of fetal aneuploidy as part of prenatal care programs also including the analyses of e.g. biochemical and ultrasound markers [2, 3]. Due to its high sensitivity and specificity screening for the common fetal trisomies 13, 18 and 21 became a widely adopted clinical application of NIPT. At first, NIPT was offered only two women with high risk for aneuploidy. Meanwhile, the limits have been widened and NIPT is offered more broadly [4]. However, up until now NIPT is still defined as a non-diagnostic screening test, because of the associated low but significant false results. Consequently, upon positive NIPT results women are counselled to consider confirmation via amniocentesis or CVS. Accordingly, one needs to take into account, that as a consequence of this routinely used recommendation a particular number of patients with a false positive NIPT result could be exposed to the miscarriage risk associated with these invasive approaches. And obviously, also false negatives results have specific consequences associated with undetected fetal mutations [5–7]. False positive results originate from a variety of different phenomenons. On the one hand they can be caused by the death of a twin in utero or true fetal mosaicism and on the other hand they may be the consequence of incidental maternal findings of e.g. specific mosaic trisomies or silent tumors. Furthermore, it has been discussed that the reliability of NIPT results also depends on the quality of the DNA in the mother’s blood, which is affected by specific medical conditions or treatments [7, 8]. False negative NIPT results, which are less common, are primarily caused by a low fetal fraction of cell-free DNA in the maternal circulation. Additionally, although NIPT is performed via the highly sensitive and specific approach of massive parallel sequencing of cffDNA, another source for false results lies within the molecular diagnostic technology itself that still harbors inherent limitations. And finally, since the placenta is considered a major source of cffDNA, confined placental mosaicism (CPM) can also cause discrepant results between NIPT and the fetal genotype. An aneuploid placenta in case of an euploid fetus can trigger a false positive result and vice versa a false negative NIPT result can originate from an euploid placenta in case of a fetal aneuploidy [3, 7–11]. In this context it is important to note that, if the placenta would not be the only major source for cffDNA, this would have implications for the interpretation of false NIPT results caused by CPM and the optimal follow-up strategy via invasive confirmation approaches in cases with positive NIPT results (see also the discussion below).

Together with cffDNA, both, fully differentiated fetal cells (including trophoblasts, nucleated erythrocytes and lymphocytes) and pregnancy-associated progenitor cells (PAPCs) in the body of pregnant women, constitute the well-known phenomenon of fetomaternal microchimerism. Due to their diverse phenotypes and plasticity, PAPCs are considered to be fetal stem cells, with characteristics and potentials between those of pluripotent stem cells and fully differentiated cells. The stemness of PAPCs is further supported by the fact that they integrate into various maternal tissues, such as e.g. blood, lung, skin, heart, spleen, liver, brain and even maternal hair follicles [12–14]. It has been demonstrated that upon maternal injury fetal microchimeric cell populations can migrate to affected maternal sites and can support tissue repair. Such a beneficial hypothesis draws a picture of protective and healing PAPCs, which might even contribute to the mother’s defense against e.g. infections or tumor development. However, it is also discussed that PAPCs could be of disadvantageous consequences being involved in maternal pregnancy conditions, such as e.g. preeclampsia [15–20]. Importantly, whereas pregnancy-associated fetomaternal microchimerism is a well-established and already intensively studied phenomenon, the origins of cffDNA and PAPCs are still a matter of debate.

Our demonstration of highly proliferative cells in human amniotic fluid expressing the stem cell marker Oct4 provided first evidence for the existence of a stem cell type nowadays known as amniotic fluid stem cells (AFSCs) [21]. AFSCs are genomically stable, mobilized fetal stem cells carrying no inherent risk of malignant transformation. They survive and persist for long time, harbor the potential to form embryoid bodies and can differentiate into cell types of all three embryonic germ layers. Upon injection into animals, AFSCs have been demonstrated to be able to integrate into different tissues and beneficially contribute to tissue regeneration [22–26]. These characteristics perfectly match and fulfill the features of PAPCs described above [12, 27]. And finally, cffDNA could be released upon apoptosis of defective or excess AFSCs, which are known to immediately undergo programmed cell death upon deregulation of their fine-tuned and strictly balanced survival control [12, 28].

Since the discovery of fetal DNA in the mother, many different sources have been discussed as primary origins: e.g. fetal DNA transferring through the amniotic membrane, the migration of free fetal DNA from fetal plasma into the maternal circulation, fetal hematopoietic cells or the liberation of fetal DNA upon destruction of fetal cells in the blood of pregnant women. In addition, besides some in vitro evidence mainly different particular in vivo observations suggest that cffDNA can derive from the placenta. Case reports on anembryonic gestations, in which only placental tissue is present, or the finding of an increase of cffDNA associated with the invasive phenotype in case reports on invasive placenta, as well as reports on pregnancies with CPM, in which the placenta has a different genotype than the fetus, indirectly support this notion [7, 8, 29–33]. Although the sum of these reports clearly underpins the widely accepted model, in which the placenta releases cffDNA into the mother’s blood as a consequence of cytotrophoblast and syncytiotrophoblast cells undergoing physiological cycles of fusion and apoptosis [3, 7], these reported findings in no way exclude the possibility that other sources also contribute to the pool of cffDNA.

Until know, the question whether other cell types can also basically function as a source of fetal DNA in the maternal circulation is not fully elucidated. We know provide evidence that human DNA can be detected in the peripheral blood of pregnant mice upon intra-amniotic injection of human AFSCs. Approximately 1% of cells in human amniotic fluid are stem cells expressing CD117 (c-Kit), the receptor for stem cell factor. Upon immunoselection through magnetic cell sorting, these human AFSCs can be isolated from native human amniotic fluid obtained via amniocentesis for routine genetic prenatal diagnosis. Human AFSCs can stably be expanded in culture in an undifferentiated status without the need for feeder layers. The used protocol and the ethical approval is described in [26]. Human AFSCs were injected into the amniotic fluid of E12.5 embryos of pregnant NOD scid gamma (NSG*™)* mice. The animal work was approved by the local ethics committee for animal care (BMBWF-68.205/0061-V/3b/2018) and was carried out in accordance with international guidelines. 24 hours after intra-amniotic injection genomic and mitochondrial DNA from the pooled injected amniotic fluids and the corresponding peripheral blood of the same mouse was isolated. Human DNA was detected via standard PCR using species-specific primers for human mitochondrially encoded cytochrome b (MT-CYB) described in [34]. In 9 peripheral blood samples of 39 mice with post-injection human DNA-positive amniotic fluid (23,1%) human MT-CYB could be detected (Fig. 1A and B). These results demonstrate that cffDNA in the maternal circulation can originate from stem cells in the amniotic fluid. In the context of this experimental approach it is important to keep in mind that the uterine structure and placentation of mice is different from humans. However, negative blood sample results could also be due to the here chosen PCR approach, that is obviously less sensitive than massive parallel sequencing routinely used for cffDNA-based NIPT in patients. However, this is the very first proof-of-principle demonstration that human AFSCs fulfill the criteria to serve as a source of fetal DNA in the mother’s blood. Whereas cell-free placental DNA in the maternal circulation is supposed to be released at the place of origin upon apoptosis of placenta cells, AFSCs, harboring many characteristics of PAPCs, could be the source of both, free fetal DNA migrating from the amniotic fluid into the maternal circulation or the liberation of fetal DNA from apoptotic AFSCs in the mother’s blood (Fig. 1C).

**Fig. 1:**
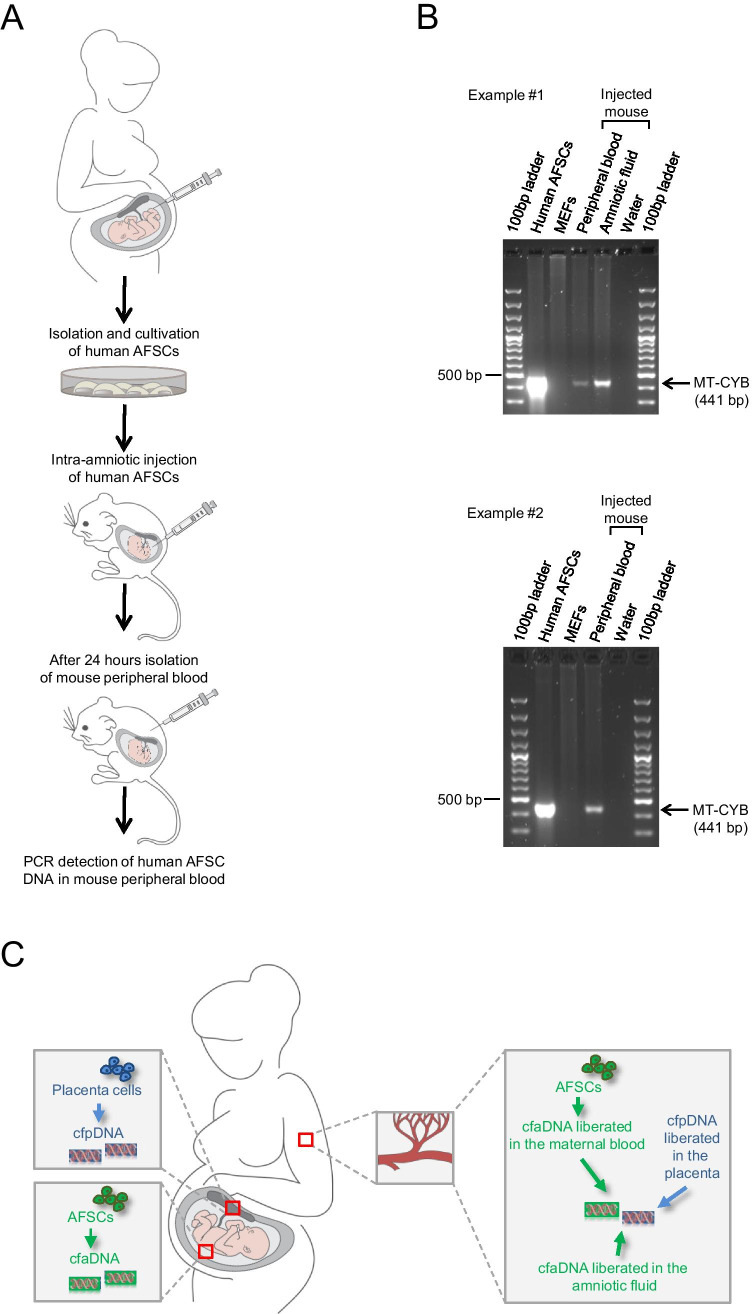
Amniotic fluid stem cells as a putative source for fetal DNA in the maternal circulation. (**A**) Illustration of the experimental approach. Human AFSCs isolated from native human amniotic fluid obtained via amniocentesis were expanded in culture. 24 hours after injection of these human AFSCs into the amniotic fluid of mouse embryos, genomic and mitochondrial DNA from the pooled injected amniotic fluids and the corresponding peripheral blood of the same mouse was isolated and human AFSC DNA was detected via PCR. (**B**) Examples #1 and #2 represent PCR analyses of human MT-CYB in different mice upon intra-amniotic injection of 2 × 10^4^ human AFSCs and 2 × 10^5^ human AFSCs, respectively. Human AFSCs, cultivated human amniotic fluid stem cells (positive control); MEFs, mouse embryonic fibroblasts (negative control). (**C**) Illustration of the contemplated model of AFSCs contributing to cffDNA. Cell-free placental DNA (cfpDNA) is liberated in the placenta from cytotrophoblast and syncytiotrophoblast cells undergoing physiological cycles of fusion and apoptosis. Cell-free amniotic fluid stem cell-derived DNA (cfaDNA) is liberated from AFSCs in the amniotic fluid. The fetal fraction of cell-free DNA in the maternal circulation is composed of cfpDNA liberated in the placenta, cfaDNA liberated in the amniotic fluid, and cfaDNA liberated from AFSCs, which crossed the fetal-maternal interface and migrated into the maternal blood

In the coming years, the general awareness of NIPT as well as the clinical experience with this screening procedure will rapidly grow [4]. Cost reduction together with the endeavor to avoid risk-carrying invasive testing procedures will trigger a continuing increase of the application of this non-invasive genetic testing approach. In this context it must be the aim of scientists, clinicians and patients alike to overcome the still existing hurdle of false NIPT results. In order to achieve this a more comprehensive picture of the cell source repertoire for NIPT will be just as indispensable as more detailed insights into the release procedures and the quantitative and qualitative composition of cffDNA. Although several independent evidences constitute the basis for the widely accepted hypothesis that placenta-derived DNA forms a relevant proportion of cffDNA analysed by NIPT [3, 7, 8, 29–33], these data do not exclude the contribution of other sources. We believe, that in the course of its foreseeable increasing routine application the continued development of NIPT from a screening approach to a diagnostic test will largely depend on the elucidation of the question whether cffDNA is exclusively of placental and not also of other fetal origin. With the here presented proof-of-principle that cffDNA could also originate from AFSCs, we want to draw the attention to this topic and to initiate further research attempts. Since it was shown that it is possible to detect an epigenetic signature of the placenta in the plasma of pregnant women [35], one could design an epigenetic analysis to investigate the proportions of cffDNA derived from placenta, AFSCs and other putative sources. Currently, the existing knowledge on the conditions or treatments affecting the quality of circulating DNA, or on the precise role of fetal conditions, of incidental maternal findings, of CPM or of low fetal DNA fractions in the maternal circulation with regard to false NIPT results, is still relatively low. It is perfectly obvious that a more detailed understanding of the cellular contributors and the putatively variable composition of cffDNA during pregnancy is an indispensable requirement for the elaboration of answers to these and many other questions. Accordingly, we believe that studies like the one presented here can contribute to both, the underlying biology of fetomaternal microchimerism and the clinical application of cffDNA.

And last but not least, guidelines regarding the optimal clinical management associated with positive NIPT results could also be affected by a more detailed elucidation of the cellular origins contributing to the composition of cffDNA. This could be of relevance to aid clinicians in advising patients to choose between follow-up testing via CVS or amniocentesis. Since NIPT is usually performed after the 10th week of gestation stressful long waiting period for parents can be avoided by CVS between 11–13 weeks of gestation instead of amniocentesis, which is usually performed between 15–18 weeks. However, if cffDNA would exclusively originate from apoptotic placenta cells, one would have to assume that due to CPM, 1–2% of NIPT results would not represent the fetus. Furthermore, NIPT should then reflect the genotype of uncultured placenta cells and consequently the analysis of direct or short-term CVS cultures would not allow the clarification of a putative CPM. Accordingly, only if both layers, the cytotrophoblast (analysed in direct or short-term cell cultures) and the mesenchymal core (investigated upon long-term CVS cultures), are found to be aneuploid, the positive NIPT result would likely represent the fetus [3, 5–7, 9]. Amniotic fluid contains AFSCs and other cells of varying origins and lineages derived e.g. from the fetal skin or the fetal urogenital, respiratory and gastrointestinal systems [22, 24]. It is important to note, that the current routine strategy to perform amniocentesis for the ultimate clarification of a putative mosaicism affecting the NIPT result would have to be reconsidered if the spectrum of sources of cffDNA would ever turn out to be more divers.

In summary, with the here addressed rationale we don't want more but also no less than to emphasize the importance of further detailed studies. It can be foreseen that more comprehensive investigations on the cellular origins of fetomaternal microchimerism will be door-openers of a fascinating stem cell research field and could pave the way to more reliable interpretations of NIPT results.

## Data Availability

The data that support the findings of this study are available on reasonable request from the corresponding author.
